# Gastric Antiulcerogenic and Hypokinetic Activities of *Terminalia fagifolia* Mart. & Zucc. (Combretaceae)

**DOI:** 10.1155/2014/261745

**Published:** 2014-05-12

**Authors:** Paulo Humberto M. Nunes, Maria do Carmo C. Martins, Rita de Cássia M. Oliveira, Mariana H. Chaves, Elcilene A. Sousa, José Roberto S. A. Leite, Leiz Maria Véras, Fernanda Regina C. Almeida

**Affiliations:** ^1^Medicinal Plants Research Center, Federal University of Piaui, 64049-550 Teresina, PI, Brazil; ^2^Department of Biophysics and Physiology, Federal University of Piaui, 64049-550 Teresina, PI, Brazil; ^3^Department of Chemistry, Federal University of Piaui, 64049-550 Teresina, PI, Brazil; ^4^Biodiversity and Biotechnology Research Center, Federal University of Piaui, 64202-020 Parnaiba, PI, Brazil; ^5^Department of Biochemistry and Pharmacology, Federal University of Piaui, 64049-550 Teresina, PI, Brazil

## Abstract

The acute toxicity, the antioxidant activity, and the pharmacological activity on the gastrointestinal tract of rodents of the ethanolic extract (TFEE) from the bark of *Terminalia fagifolia* Mart. & Zucc. (Combretaceae) and of its aqueous (TFAqF), hydroalcoholic (TFHAF), and hexanic (TFHEXF) partition fractions have been evaluated. TFEE presented low acute toxicity, antioxidant, and antiulcerogenic activity against ethanol-induced ulcers, which was partially blocked by pretreatment with L-NAME and indomethacin. It reduced the total acidity and raised the pH of gastric secretion. Additionally, TFEE delayed gastric emptying and slightly inhibited the small intestinal transit and also presented a weakly antidiarrheal activity. The antiulcerogenic and antioxidant activity were also detected in TFAqF and TFHAF but not in TFHEXF. The antisecretory and gastroprotective activity of TFEE partially involve the nitric oxide and prostaglandin participation. Nevertheless, TFEE, TFAqF, and TFHAF drastically reduced the mucus layer adhered to the gastric wall of rats treated with ethanol or indomethacin. Complementary studies are required in order to clarify the paradox of the presence of a gastroprotector activity in this plant that, at the same time, reduces the mucus layer adhered to the gastric wall.

## 1. Introduction 

Medicinal plants are used and marketed worldwide as herbal drugs or as single active ingredients over centuries. Besides their popular consumption to treat and cure human illness, plant derived natural products can play an important role as a source of pharmacologic tools to enable the understanding of the biochemical pathways and mechanisms of disease [1, 2].

In Brazil, medicinal plants are widely used in both rural and urban areas. Most are used according to folk tradition developed by natives or brought to the country by Europeans, Africans, and Asians [3]. Considering the cultural and economic perspectives, a scientific verification of plant use is important for the assessment of their quality, safety, and therapeutic efficacy.

*Terminalia fagifolia* Mart. & Zucc. (Combretaceae), commonly known as “chapadeiro,” is a medicinal plant used in traditional folk medicine for its effective treatment of gastrointestinal disturbances, such as ulcer, gastritis, and diarrhea [4]. Diarylpropanes, flavanones, chalcones, flavan, gallic acid, and sitosterol were isolated from the heartwood and trunk bark of* T. fagifolia* and were evaluated for their* in vitro *cytotoxic activity against two human cancer cell lines and antioxidant properties [5]. Ayres et al. [6] reported the isolation of (+)-catechin, sitosterol-3-*O*-*β*-D-glucopyranoside, *α*- and *β*-tocopherol, a mixture of lupeol, *α*- and *β*-amyrin, sitosterol, and a mixture of glycosidic flavonoids in the ethanolic extract of the leaves of* T. fagifolia* and investigated the antioxidant activity of fractions of this extract.

Literature about the botanical family Combretaceae reports the detection of a variety of biological activities and a ubiquitous antioxidant property in genuses* Terminalia* and* Combretum* [7–21]. Investigations with* Terminalia arjuna* (Roxb.) Wight & Arn. [22, 23],* Terminalia avicennioides* [24],* Terminalia bellirica* (Gaertn.) Roxb. [25, 26],* Terminalia brasiliensis* [27],* Terminalia catappa* [28],* Terminalia chebula* Retz. [26, 29–31],* Terminalia pallida* Brandis [32],* Combretum leprosum* Mart. & Eiche [33],* Combretum dolichopetalum* Engl. & Diels [34], and* Guiera senegalensis* J.F. Gmel [35] led to interesting results when they focused on the antiulcerogenic activity. The ethanolic extract of* Combretum dolichopetalum* Engl. & Diels also delayed gastric emptying in rats [34]. The methanolic bark extract of* Terminalia arjuna* showed a significant increase in the adherent mucus of the gastric wall and in the protein bound carbohydrate complexes of the gastric juice in rats treated with diclofenac sodium [22].

Gastric ulcer is one of the most common gastrointestinal diseases and results from an imbalance between the action of aggressive and defensive factors on the gastric mucosa [36]. The defense mechanisms are crucial for the maintenance of an effective barrier and are arranged at different levels, which work together to protect the stomach. The preepithelial level or the first line of defense consists of the mucus layer and bicarbonate secreted into the mucus, creating a pH gradient within the mucus. The epithelial level consists of intercellular tight junctions and proton and bicarbonate transport systems. The postepithelial level consists mainly of an effective blood flow and the gastrointestinal autonomic nervous system [37]. Gastric mucosal damage can be induced by aggressive factors like hydrochloric acid, pepsin, leukotrienes, free radicals, nonsteroidal anti-inflammatory drugs (NSAIDs), ischemia, dysmotility, ethanol, nicotine, and stress [38].

Despite the popular use of* Terminalia fagifolia* as a medicinal plant, there are few data about its pharmacological effect, particularly on the gastrointestinal tract. In order to validate the ethnopharmacological uses of these natural products, the present study has been conducted to evaluate the acute toxicity and the antioxidant and pharmacological activity of the ethanolic extract from the stem bark of* Terminalia fagifolia* (TFEE) and of its partition fractions on the gastrointestinal tract of rodents. Additionally, the total phenolic and flavonoid content of the extract and partition fractions has been determined.

## 2. Material and Methods

### 2.1. Animals

Male (246 ± 5 g) and female (164 ± 6 g) Wistar rats and male (27 ± 1.2 g) and female (24 ± 0.8 g) Swiss mice were used for the study. The animals were provided with a rodent-pellet diet (LABINA 5002, EVIALIS do Brasil Nutrição Animal Ltda., Sao Paulo, Brazil) and water* ad libitum*. They were maintained in proper conditions, temperature of 25 ± 2°C, approximately 60% humidity, and 12 h light/dark cycles. The animals were randomly assigned to different control and treatment groups. The experimental protocols were conducted with 6 to 8 animals/group in accordance with the guidelines of the Brazilian Council of Animal Experimental Control and approved by the Ethics Committee for Animal Research at the Federal University of Piaui (Protocol number 042/09). 

### 2.2. Plant Material and Extracts Preparation

The stem barks of* Terminalia fagifolia* Mart. & Zucc. (Combretaceae) were collected in November 2006 at the “Bambu” community, Timon-MA, Brazil. A voucher specimen (TEPB number 21.691) was deposited in the Graziela Barroso Herbarium at the Federal University of Piaui, Teresina, state of Piaui, Brazil. The plant material was shade-dried at approximately 40°C and the stem bark powder was exhaustively extracted with 99.6% ethanol or 50% hydroalcoholic solution at room temperature. After filtration, the solvents were eliminated in a vacuum at 50°C and the concentrates were lyophilized to obtain the dry* Terminalia fagifolia* ethanolic (TFEE) and hydroalcoholic (TFHAE) extracts which were stored under refrigeration until further use. For the experiments these extracts were freshly prepared as a suspension in distilled water.

### 2.3. Preparation of the Partition Fractions of TFEE

To obtain the partition fractions of TFEE, it was formerly dissolved in a methanol/distilled water solution (1 : 2) and extracted with ethyl acetate. In sequence, the ethyl acetate phase was concentrated and dissolved in a methanol/distilled water solution (9 : 1) and extracted with hexane. The phases obtained were concentrated by elimination of the solvents and resulted in the aqueous fraction (TFAqF), hydroalcoholic fraction (TFHAF), and hexane fraction (TFHEXF) of TFEE.

### 2.4. Chromatographic Analysis of TFEE and Its Partition Fractions

TFEE and its partition fractions (TFAqF, TFHAF, and TFHEXF) were analyzed by thin layer chromatography (TLC) and high performance liquid chromatography (HPLC).

The TLC analysis of TFEE, TFAqF, TFHAF, and TFHEXF was performed using silica gel plates on glass (2.5 cm × 6 cm) developed with three solvent mixtures composed of hexane/acetyl acetate (8 : 2), chloroform/methanol (9 : 1), or chloroform/methanol/water (65 : 30 : 5). The spots on the TLC plates were revealed with cerium sulfate spray solution followed by heating at 100°C for 5 minutes [39].

For HPLC analysis, aliquots of TFEE, TFAqF, and TFHAF were diluted in ethanol and water (8 : 2) and filtered with 0.45 *μ*m membranes. The samples were injected in high performance liquid chromatography (SHIMADZU Prominence, AUTOSAMPLER SIL-10AF, CTO-20A, LC-6AD, CBM-20A, and SPD-20A). The column used was RP C18 (4.6 × 250 mm, i.d. 5 *μ*m Phenomenex Luna, USA) and the mobile phase consisted of formic acid 2% (v/v) and acetonitrile doped with 0.1% of trifluoroacetic acid (TFA) (v/v), starting with a linear gradient elution of 0–100% in 40 min. Flow rate was 1 mL/min and sample injection was 40 *μ*L. The effluent was monitored at 276 nm with a UV-VIS detector. Samples of (+)-catechin and (−)-epicatechin were prepared under the same conditions and were used as standard.

### 2.5. Determination of the Antioxidant Activity by the DPPH Free Radical Scavenging Assay

The free radical scavenging activity was measured using the 2,2-diphenyl-1-picrylhydrazyl (DPPH) assay [19] for the determination of the stoichiometry of the reactions (static version) and for the characterization of the reactivity of each sample (dynamic version). For the static version a stock solution (1 mg/mL) of TFEE, TFAqF, TFHAF, or TFHEXF was diluted to final concentrations of 240, 120, 60, 30, 15, and 5 *μ*g/mL, in methanol. Two hundred *μ*L of the solutions with different concentrations was added to 2 mL of 40 *μ*g/mL DPPH methanol solution and allowed to react at room temperature. After 30 min the absorbance values were measured at 516 nm by a UV-VIS spectrophotometer (Biospectro SP-220, EQUIPAR Ltda., Curitiba, Brazil) and used to calculate the percentage of the antioxidant activity (AA%) and the EC_50_ (efficient concentration = the concentration of the antioxidant necessary to decrease the initial DPPH concentration by 50%). The reactivity of each sample was evaluated at 60 *μ*g/mL concentration by the measurement of the decrease in the absorbance at 516 nm for 60 min. The values of the absorbance were used to calculate the percentage of the remaining DPPH at each 20 s of interval and the ET_50_ (efficient time = the time necessary to decrease the initial DPPH concentration by 50%).

The antioxidant activity (AA%) was calculated using the following formula: AA% = [(absorbance of the control + absorbance of the blank − absorbance of the sample)/absorbance of the control] × 100. Methanol (2 mL) plus plant extract solution (200 *μ*L) was used as a blank. DPPH solution (2 mL) plus methanol (200 *μ*L) was used as a negative control. Catechin and butylated hydroxytoluene (BHT) were used as the standard solutions. Assays were carried out in triplicate. The EC_50_ and ET_50_ values were calculated by nonlinear regression.

### 2.6. Determination of the Total Phenolic and Flavonoid Content

Total phenolic content (TPC) was determined spectrophotometrically by the Folin-Ciocalteu method [40]), with minor adaptation. Briefly, reaction medium contains 2 mL of distilled water, 250 *μ*L of Folin-Ciocalteu reagent, and 250 *μ*L of extract or fraction (200 *μ*g/mL). After 5–8 min in the dark, 100 *μ*L of 10% Na_2_CO_3_ solution was added and mixed. The mixture was incubated for one hour at room temperature (24°C) in the dark and the absorbance was measured at 760 nm. Gallic acid (10–160 *μ*g/mL) was used to construct a standard curve (*Y* = 0.0046*X* − 0.0246;  *r* ^2^ = 0.9994). The results were expressed as mg of gallic acid equivalent (GAE)/g dry weight. All tests were performed in triplicate.

Total flavonoid content (TFC) was determined according to Woisky and Salatino [41], with minor modifications. Using stopped glass tubes, 1000 *μ*L of samples (500 *μ*g/mL, in ethanol) was mixed with an equal volume of 2% AlCl_3_ in ethanol. After one hour at room temperature (24°C) in the dark, the absorbance was measured at 420 nm. A standard curve (*Y* = 0.0053*X* + 0.0043; *r* ^2^ = 0.9999) was constructed with rutin (10–160 *μ*g/mL) and the total flavonoid content was expressed as mg rutin equivalent (RE)/g dry weight. Samples were analyzed in triplicate.

### 2.7. Evaluation of the Acute Oral Toxicity in Mice

This assay was performed according to the Organization for Economic Cooperation and Development (OECD) revised up-and-down procedure for acute toxicity testing [42] in groups of 5 male and 5 female Swiss mice. The animals were fasted overnight (12 h) with free access to water prior to the oral administration of a single dose of 2000 mg/kg of TFEE, TFAqF, or TFHAF and observed continuously for 4 h, intermittently for 24 h, and then once a day for the next 14 days for general behavioral changes, signs of toxicity, and mortality.

### 2.8. Acute Gastric Ulcer Induced by Ethanol in Rats

The antiulcerogenic activity of TFEE (60.5, 125, 250, or 500 mg/kg) and TFHAE, TFAqF, TFHAF (as a suspension in distilled water), TFHEXF (dissolved in Tween 80 1%), or carbenoxolone at dose 250 mg/kg (in distilled water) was investigated by using the acute ethanol-induced gastric ulcer in rats, adapted from Robert et al. [43].

Male rats maintained under standard conditions as described above were fasted for 24 h and orally received distilled water (control, 5 mL/kg) or vehicle (Tween 80 1%, 5 mL/kg), the sample to be evaluated (test), or carbenoxolone (standard). One hour later, gastric lesions were induced by oral administration of absolute ethanol (1 mL/animal). Animals were euthanized 30 min after ethanol administration with sodium thiopental overdose (100 mg/kg, i.p.) and the stomachs were removed, opened along the lesser curvature, washed with normal saline, and examined in a blinded manner. The quantification of the ulceration induced by ethanol was performed using the* ImageJ*-NIH^R^ computer program (National Institutes of Health, Washington D.C.) to calculate the ulceration index expressed as the percentage of ulcerated area in relation to the area of the corpus of the stomach.

### 2.9. Pretreatment with Indomethacin on Ethanol-Induced Gastric Ulcer in Rats

Male rats, maintained under standard conditions as described previously, were fasted for 24 h with free access to water then divided into four groups according to the respective treatment. The animals were administered a subcutaneous injection of indomethacin (30 mg/kg, s.c.), a cyclooxygenase (COX) inhibitor. After 30 min, each group received the respective treatment orally (distilled water, 250 or 500 mg/kg of TFEE, or 250 mg/kg of carbenoxolone). One hour later, 1 mL/animal of absolute ethanol was administered orally. The stomachs were removed 30 min after ethanol administration. The gastric mucosal lesions were evaluated and the ulceration index was calculated as described in Section 2.8.

### 2.10. Pretreatment with L-NAME on Ethanol-Induced Gastric Ulcer in Rats

Male rats, maintained under standard conditions as described previously, were fasted for 24 h with free access to water and then divided into five groups according to the respective treatment. The animals were administered an injection of N-nitro-L-arginine methyl ester (L-NAME, 70 mg/kg, i.p.), a nitric oxide synthase (NOS) inhibitor. After 30 min, each group received the respective oral treatment (distilled water, 250 or 500 mg/kg of TFEE, or 100 or 250 mg/kg of carbenoxolone). One hour later, 1 mL/animal of absolute ethanol was administered orally. The stomachs were removed 30 min after ethanol administration and the gastric mucosal lesions were evaluated for the quantification of the ulceration index as described in Section 2.8.

### 2.11. Determination of the Gastric Juice Volume and Acid Secretion in Pylorus Ligated Rats

Female rats were acclimatized under standard conditions as described above for at least 4 days in individual, metabolic, wire-bottom cages to avoid coprophagy. The food was withdrawn 24 h before the experiment but there was free access to drink a 5% glucose solution to reduce fasting stress. The control and experimental groups consisted of 6–8 animals each. All experiments were done in the morning. Pylorus ligation was performed as described by Shay et al. [44] and was done through a midline abdominal incision under ketamine (50 mg/kg, i.m.) and xylazine (10 mg/kg, i.m.) anesthesia. TFEE was administered intraduodenally to the animals in a 500 mg/kg dose suspended in 5 mL/kg of distilled water. Control animals received distilled water (5 mL/kg) and the standard group received ranitidine (60 mg/kg). The abdomen was sutured and the animals were allowed to recover from anesthesia. Rats were euthanized with sodium thiopental (100 mg/kg, i.p.) 4 h after treatment and the abdomen was opened and another ligature was placed around the esophagus close to the diaphragm. The stomachs were removed and gastric juice solution was collected. Distilled water (3 mL) was added and the total solution was centrifuged at 3500 rpm for 30 min. The content (mL), the pH, and the total acidity in gastric secretion were determined in the supernatant volume. The total acidity output was determined by titration to pH 7.4 with 0.1 N NaOH in a pH meter (WTW 330i, Wissensctlich-Technische Werkstätten GmbH & Co. KG, Weilheim, Germany) and expressed as *μ*Eq/h gastric juice. Additionally, glandular segments from the stomachs were excised for determination of gastric wall mucus and nonprotein sulfhydryl (NP-SH) group content, as described in Sections 2.12 and 2.13.

### 2.12. Determination of the Gastric Wall Mucus Content in Pylorus Ligated Rats

Gastric wall mucus was assessed by the Alcian blue method [45]. Stomachs excised from 4 h pylorus ligated rats were opened along the lesser curvature. Glandular segments from the stomachs were removed and weighed. Each segment was transferred immediately to 7 mL of 0.25% w/v Alcian blue solution (0.16 M sucrose in 0.05 M sodium acetate, pH 5.8) and incubated for 2 h at room temperature. The free dye was removed by two successive rinses at 15 and 45 min in 0.25 M aqueous sucrose solution. The gastric wall mucus bound dye was extracted by immersion in 5 mL of 0.5 M MgCl_2_ for 2 h with 1 min agitation every 30 min. A 4 mL sample of the blue extract was then shaken vigorously with an equal volume of diethyl ether and the resulting emulsion was centrifuged at 3000 rpm for 10 min. The optical density of Alcian blue in the aqueous layer was read against distilled water at 598 nm by a UV-VIS spectrophotometer (Biospectro SP-220, EQUIPAR Ltda., Curitiba, Brazil). The quantity of mucin was expressed as *μ*g of Alcian blue extracted per weight (g) of wet stomach glandular tissue.

### 2.13. Determination of the Gastric Wall Nonprotein Sulfhydryl Group Content in Pylorus Ligated Rats

Gastric wall nonprotein sulfhydryl (NP-SH) groups were determined by the method from Sedlak and Lindsay [46]. Stomachs excised from 4 h pylorus ligated rats were opened along the lesser curvature. Glandular segments from the stomachs were removed and weighed. Each segment was transferred immediately and homogenized in 5 mL of refrigerated 0.02 M sodium EDTA (ethylenediaminetetraacetic acid, disodium salt) solution. Tissue proteins (in 4 mL homogenate) were precipitated with 4 mL of 10 g% trichloroacetic acid and centrifuged out (15 min, 3000 rpm) and an aliquot (2 mL) of the supernatant was added to 4 mL of 0.4 M Tris/0.2 M EDTA pH 8.9 and 100 *μ*L of 0.01 M DTNB (5,5′-dithiobis(2-nitrobenzoic acid)), diluted in methanol. The optical density of the TNB (thionitrobenzoic) ion solution was read against distilled water at 412 nm by a UV-VIS spectrophotometer (Biospectro SP-220, EQUIPAR Ltda., Curitiba, Brazil) and the concentration of sulfhydryl group was calculated by comparison to a standard calibration curve prepared with cysteine. The content of NP-SH group was expressed as *μ*M SH per weight (g) of wet stomach glandular tissue.

### 2.14. Determination of the Gastric Wall Mucus and Nonprotein Sulfhydryl Group Contents in Rats Treated with Ethanol

Female rats, maintained under standard conditions as described previously, were fasted for 24 h with free access to water then divided into groups according to the respective treatment. The animals orally received distilled water (5 mL/kg, control groups with and without ethanol aggression), TFEE (500 mg/kg, test groups with and without ethanol aggression), TFAqF (250 mg/kg), TFHAF (250 mg/kg), carbenoxolone (250 mg/kg), or N-acetylcysteine (500 mg/kg). One hour later, gastric aggressive lesions were induced by oral administration of absolute ethanol (5 mL/kg). Animals were euthanized 30 min after ethanol administration with sodium thiopental overdose (100 mg/kg, i.p.) and the stomachs were removed, opened along the lesser curvature, and softly washed with normal saline. Glandular segments from the stomachs were removed and weighed and the gastric wall mucus and nonprotein sulfhydryl group contents were determined as described in Sections 2.12 and 2.13.

### 2.15. Determination of the Gastric Wall Mucus and Nonprotein Sulfhydryl Group Contents in Rats Treated with Indomethacin

Male rats maintained under standard conditions as described previously, were fasted for 24 h with free access to water then divided into groups according to the respective treatment. The animals orally received distilled water (5 mL/kg, control groups with and without indomethacin aggression), TFEE (500 mg/kg), TFAqF (250 mg/kg), TFHAF (250 mg/kg), or carbenoxolone (250 mg/kg). Thirty minutes later, gastric aggressive lesions were induced by subcutaneous administration of indomethacin (30 mg/kg). Animals were euthanized four hours after indomethacin administration with sodium thiopental overdose (100 mg/kg, i.p.) and the stomachs were removed, opened along the lesser curvature, and softly washed with normal saline. Glandular segments from the stomachs were removed and weighed and the gastric wall mucus and nonprotein sulfhydryl group contents were determined as described in Sections 2.13 and 2.14.

### 2.16. Assessment of the Gastric Emptying and Bowel Transit in Rats

The gastric emptying and small intestinal transit were assessed by the phenol red content assay, modified from the method described by Izbeki et al. [47]. Briefly, groups of 6–8 female rats were fasted for 24 h and orally received water (5 mL/kg), TFEE (250 or 500 mg/kg), TFAqF (250 mg/kg), TFHAF (250 mg/kg), or atropine (3 mg/kg, i.p.). One hour later, they all orally received phenol red 0.5 mg/mL in glucose 5 g% (1.5 mL/animal). After 20 min, the animals were euthanized with an overdose of sodium thiopental (100 mg/kg, i.p.) and the stomach and small intestine were removed. The small intestine was divided into the proximal (40%), medial (30%), and distal (30%) portions and each segment was homogenized in 100 mL of 0.1 N NaOH. Tissue proteins (in 5 mL homogenate) were precipitated with 0.5 mL of 20 g% trichloroacetic acid and centrifuged out (20 min, 3000 rpm). From the supernatant, an aliquot of 3 mL was added to 4 mL of 0.5 N NaOH and the concentration of phenol red was determined by absorbance at 560 nm (Biospectro SP-220 UV-VIS spectrophotometer, EQUIPAR Ltda., Curitiba, Brazil). The content of the dye in each segment was calculated and the retention of the marker was expressed as the percentage of the total amount of phenol red recovered in the four segments.

### 2.17. Small Intestinal Transit in Mice

Male and female mice, fasted for 24 h, were orally administered distilled water (10 mL/kg), TFEE (500, 750, or 1000 mg/kg), TFHAE (500, 750, or 1000 mg/kg), or atropine sulfate (3 mg/kg) and 30 min later individually received 0.1 mL of a 10% aqueous suspension of charcoal meal. Half an hour after this treatment, each animal was euthanized with a sodium thiopental overdose (100 mg/kg, i.p.) and the intestinal transit of the meal was evaluated by the measurement of the distance travelled in 30 min by the charcoal from the pylorus to the caecum and expressed as the percentage of the full small intestinal length.

### 2.18. Castor Oil-Induced Diarrhea in Mice

Male and female mice, fasted for 20 h, were divided into six groups of 8 animals each. The first group was orally administered distilled water while the other groups received castor oil (0.1 mL/animal). Half an hour later, the animals orally received distilled water (first and second groups, 10 mL/kg), TFEE (500, 750, or 1000 mg/kg), or loperamide (3.5 mg/kg) and were placed separately in plastic cages with paper sheets. The paper sheet was changed and the number of compact and diarrheal faeces excreted for each animal was scored every hour for 4 h. The severity of the diarrhea was assessed by the total number of compact and diarrheal faeces excreted by each group of animals in the 4 h interval time of observation.

### 2.19. Statistical Analysis

The results are presented as the mean ± standard error of the mean (M ± S.E.M). The statistical significance for differences between groups was calculated by one-way analysis of variance (ANOVA) and Dunnett's or Tukey's test. The differences between groups were regarded as significant at *P* < 0.05.

## 3. Results

### 3.1. Preparation of the Extracts and Partition Fractions

The extraction of the* Terminalia fagifolia* bark powder (630 g) with ethanol (6 L) rendered 120 g (19% yield) of TFEE. Similar procedure using 60 g of bark powder extracted with 900 mL of a 50% hydroalcoholic solution as solvent resulted in 18 g (30% yield) of TFHAE.

The solvent extraction of TFEE (80 g) with acetyl acetate and hexane produced the partition fractions TFAqF (26.8 g, 33.5% yield), TFHAF (43.45 g, 54.3% yield), and TFHEXF (1.7 g, 2.1% yield).

### 3.2. Thin Layer Chromatography (TLC) and High Performance Liquid Chromatography (HPLC) of TFEE, TFAqF, and TFHAF

The thin layer chromatograms, developed on silica gel plates with three solvent mixtures, suggested the presence of polar compounds like flavonoids, glycosylated flavonoids (yellow spots), and saponins (rose spots) in the aqueous (TFAqF) and hydroalcoholic (TFHAF) partition fractions and fat substances, including steroids (blue spots which become pink and then gray with the extension of the heating) and pentacyclic triterpenoid (rose and orange spots), in the hexanic (TFHEXF) partition fraction of TFEE.

**Figure 1:**
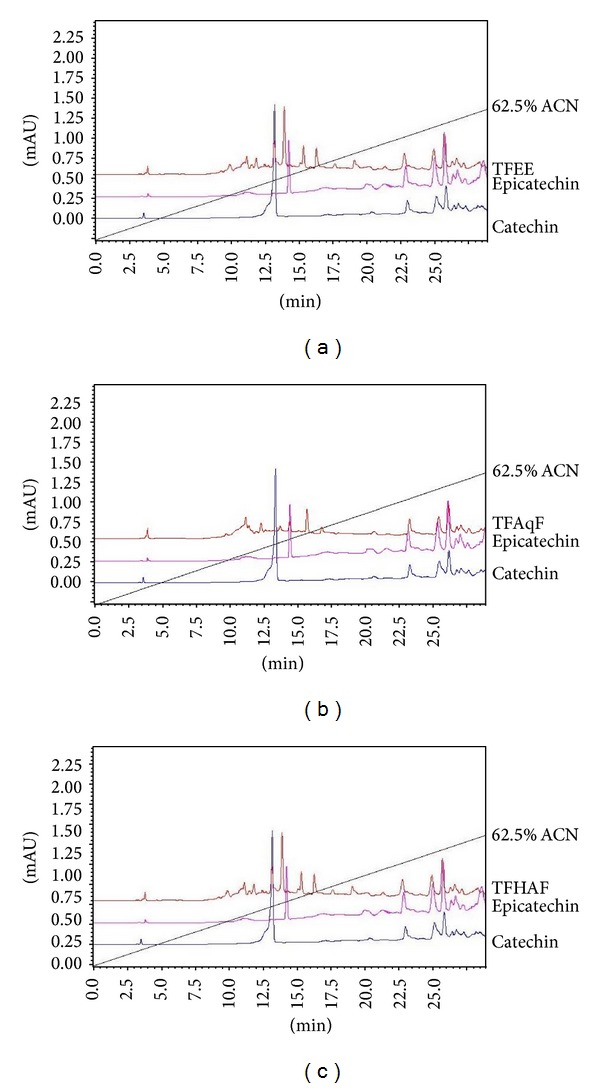
HPLC chromatograms of the ethanolic extract of the bark of* Terminalia fagifolia* (TFEE (a)) and of the aqueous (TFAqF (b)) and hydroalcoholic (TFHAF (c)) partition fractions of TFEE. Gradient elution (0–100%) using formic acid at 2% and acetonitrile doped with 0.1% of TFA on the C18 RP column, with flow rate of 1 mL/min, monitored at 276 nm using UV-VIS detector. Standards: (+)-catechin and (−)-epicatechin.

The chromatograms of the HPLC analysis of TFEE, TFAqF, and TFHAF are depicted in Figure 1. The results allowed the identification of components with retention time similar to (+)-catechin and (−)-epicatechin in TFEE and TFHAF and to (−)-epicatechin in TFAqF.

### 3.3. Antioxidant Activity by the DPPH Free Radical Scavenging Assay

The results of the study of the DPPH free radical scavenging activity of TFEE, partition fractions of TFEE (TFAqF, TFHAF, and TFHEXF), catechin, and BHT are presented in Table 1. TFEE showed an EC_50_ equivalent to that of catechin and much lower (*P* < 0.001) than that of BHT and of TFHEXF. There was no statistical difference between the EC_50_ of TFEE and its aqueous or hydroalcoholic fractions. The hexanic fraction of TFEE (TFHEXF) showed an EC_50_ higher than that of BHT. Nevertheless, the reactivity (ET_50_) of TFEE, TFAqF, and TFHAF was higher (*P* < 0.001) than that of catechin at 60 *μ*g/mL. The reactivity of BHT and TFHEXF was not quantified because of the higher EC_50_ of these samples.

### 3.4. Total Phenolic and Flavonoid Content

Table 1 shows the total phenolic (TPC) and flavonoid (PFC) content of TFEE and its partition fractions, catechin, and butylated hydroxytoluene (BHT). There was no significant difference between the TPC (mg gallic acid equivalent/g) of TFAqF (400.5 ± 9.1) and TFHAF (404.1 ± 12.9), which was about 11% lower than that of TFEE (452.3 ± 18.1). The TFC (mg rutin equivalent/g) of TFEE (218.6 ± 2.0), TFAqF (217.5 ± 1.6), and TFAHF (222.0 ± 1.9) was similar. TFHEXF showed a very low TPC (76.6 ± 1.3) and TFC (7.4 ± 0.8), compared to that of TFEE (17% and 3%, resp.).

### 3.5. Acute Oral Toxicity Evaluation

The result of the toxicity study of TFEE, TFAqF, and TFHAF at a limit oral test dose of 2000 mg/kg in male and female mice is shown in Table 2. According to the OECD revised up-and-down procedure for acute toxicity testing [42], the LD_50_ of TFEE, TFAqF, and TFHAF is greater than 2000 mg/kg for mice with a gender difference in the acute toxicity of TFEE where the females are more sensitive than the males and could be classified as of low acute toxicity hazard category 5 according to the United Nations Globally Harmonized System of Classification and Labeling of Chemicals [48].

### 3.6. Effect of TFEE on Acute Gastric Ulcer Induced by Ethanol in Rats

**Figure 2:**
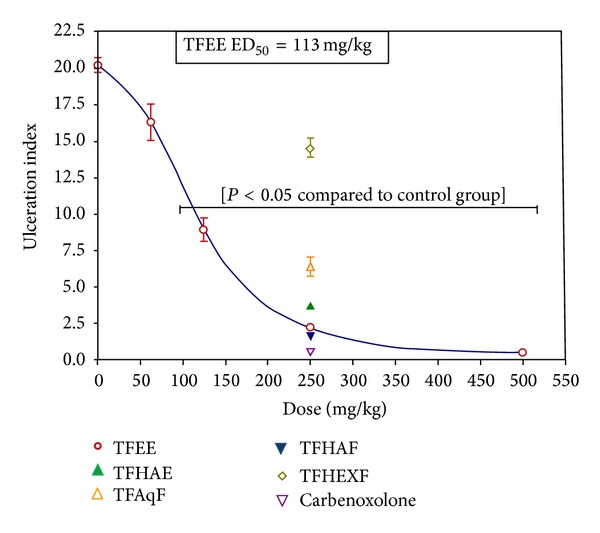
Effect of the oral administration of the ethanolic (TFEE, 0–500 mg/kg) and hydroalcoholic (TFHAE, 250 mg/kg) extracts of the bark of* Terminalia fagifolia* and of the aqueous (TFAqF), hydroalcoholic (TFHAF), and hexanic (TFHEXF) partition fractions of TFEE or carbenoxolone (250 mg/kg) on gastric ulcers induced by absolute ethanol. Ulceration index as percentage of the area of the corpus of the stomach (data are presented as the mean ± S.E.M). *P* < 0.05 compared to the control group (ANOVA and Dunnett's test).

In the ethanol-induced gastric ulcer model, TFEE was found to possess remarkable ulcer-protective properties at orally administered doses of 125, 250, and 500 mg/kg, showing 56%, 89%, and 97% protection, respectively. The antiulcerogenic activity of TFEE was dose-related, showing an effective dose (ED_50_) of 113 mg/kg. Inhibition of the gastric ulcerogenic activity of ethanol was also detected in TFAqF (68%), TFHAF (92%), and TFHAE (81%), but not in TFHEXF, at orally administered doses of 250 mg/kg. The standard drug carbenoxolone (250 mg/kg) showed 97% protection (Figure 2).

### 3.7. Effects of TFEE on Acute Gastric Ulceration Induced by Ethanol with Pretreatment of Indomethacin

**Figure 3:**
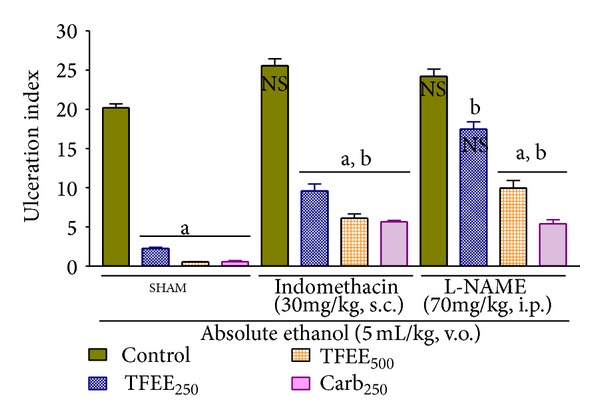
Effect of the oral administration of the ethanolic extract of the bark of* Terminalia fagifolia* (TFEE, 250 and 500 mg/kg) or carbenoxolone (250 mg/kg) on gastric ulcers induced by absolute ethanol in the absence (SHAM) or presence of pretreatment with L-nitroarginine-methyl-ester (L-NAME) or indomethacin in rats. Ulceration index as percentage of the area of the corpus of the stomach (data are presented as the mean ± S.E.M.). ^NS^ *P* > 0.05 compared to SHAM control group. ^a^ *P* < 0.05 compared to the respective control group (ANOVA and Dunnett's test). ^b^ *P* < 0.01 compared to the SHAM group treated with TFEE or carbenoxolone at the same dose (ANOVA and Tukey's test).

Pretreatment with the cyclooxygenase inhibitor indomethacin (30 mg/kg, s.c.) weakened the protection against gastric ulceration induced by ethanol from 89% to 52% for TFEE at the 250 mg/kg dose, from 97% to 70% for TFEE at the 500 mg/kg dose, and from 97% to 72% for carbenoxolone at the 250 mg/kg dose (Figure 3).

### 3.8. Effects of TFEE on Acute Gastric Ulceration Induced by Ethanol with Pretreatment of L-NAME

The protection against gastric ulceration induced by ethanol was weakened by the pretreatment with the nitric oxide synthase inhibitor L-NAME (70 mg/kg, i.p.) from 97% to 51% for TFEE at 500 mg/kg and from 97% to 73% for carbenoxolone at 250 mg/kg dose and it abolished the gastroprotection shown by TFEE at 250 mg/kg dose (Figure 3).

### 3.9. Effect of TFEE on Gastric Acid Secretion and on Gastric Wall Mucus and Nonprotein Sulfhydryl Group Contents in Pylorus Ligated Rats

Intraduodenal treatment with 500 mg/kg of TFEE in four-hour pylorus ligated female rats provoked a significant (*P* < 0.05) increase in pH and a reduction in the volume and total acidity of gastric juice produced. The response elicited by 60 mg/kg of ranitidine was similar to that obtained with TFEE. However, ranitidine's effect on the total acidity and pH was significantly greater than those of TFEE (Table 3). The intraduodenal administration of TFEE or ranitidine did not result in any significant change in the mucus or NP-SH group contents of the animals' gastric mucosa.

### 3.10. Effect of TFEE, TFAqF, and TFHAF on Gastric Wall Mucus and Nonprotein Sulfhydryl Group Contents in Rats Treated with Ethanol

**Figure 4:**
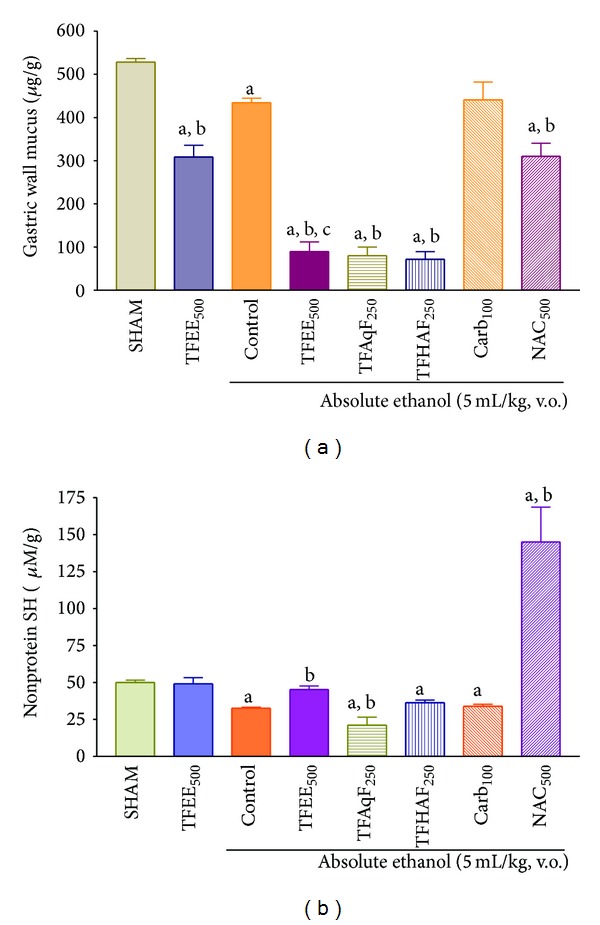
Effect of the oral administration of the ethanolic extract (TFEE) of the bark of* Terminalia fagifolia *(500 mg/kg) or its aqueous (TFAqF) and hydroalcoholic (TFHAF) partition fractions (250 mg/kg), carbenoxolone (Carb, 100 mg/kg), or N-acetylcysteine (NAC, 500 mg/kg) on the gastric wall mucus (a) and on the nonprotein sulfhydryl (NP-SH) group content (b) in female rats orally treated with absolute ethanol. The data represent the mean ± S.E.M. of 8 animals/group. *P* < 0.01 (ANOVA and Dunnett's test) compared to SHAM (^a^), control group (^b^), or TFEE without ethanol treatment (^c^).

The effects of the oral administration of the ethanolic extract (TFEE) of the bark of* Terminalia fagifolia *(500 mg/kg) or its aqueous (TFAqF) and hydroalcoholic (TFHAF) partition fractions (250 mg/kg), carbenoxolone (100 mg/kg), or N-acetylcysteine (500 mg/kg) on the gastric wall mucus and nonprotein sulfhydryl (NP-SH) group contents in female rats orally treated with absolute ethanol are presented in Figure 4. TFEE, TFAqF, and TFHAF drastically reduced (*P* < 0.01) the gastric wall mucus content of the animals treated with ethanol, and the effect of TFEE was present even in the absence of this aggressive agent. TFEE by itself did not change the gastric wall nonprotein sulfhydryl (NP-SH) group level but restored it in the presence of the aggressive agent. TFHAF had no effect and TFAqF showed an effect similar to that of ethanol, reducing significantly (*P* < 0.01) the gastric wall nonprotein sulfhydryl (NP-SH) group content of the animals treated with ethanol.

### 3.11. Effect of TFEE, TFAqF, and TFHAF on Gastric Wall Mucus and Nonprotein Sulfhydryl Group Contents in Rats Treated with Indomethacin

**Figure 5:**
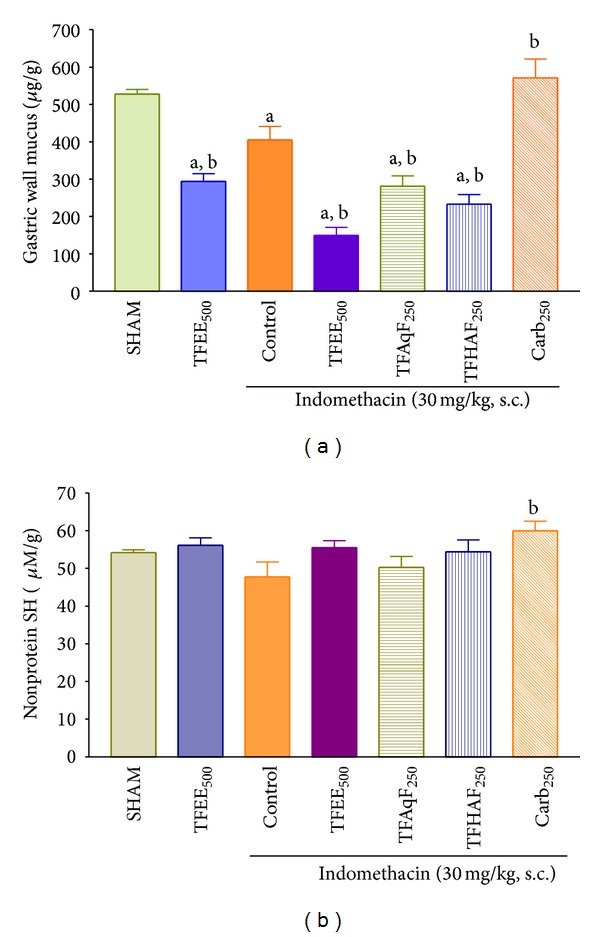
Effect of the oral administration of the ethanolic extract (TFEE) of the bark of* Terminalia fagifolia *(500 mg/kg) or its aqueous (TFAqF) and hydroalcoholic (TFHAF) partition fractions (250 mg/kg) or carbenoxolone (Carb, 250 mg/kg) on the gastric wall mucus (a) and on the nonprotein sulfhydryl (NP-SH) group content (b) in male rats treated by subcutaneous route with indomethacin. The data represent the mean ± S.E.M. of 8 animals/group. *P* < 0.01 (ANOVA and Dunnett's test) compared to SHAM (^a^) or control group (^b^).

The effects of the oral administration of the ethanolic extract (TFEE) of the bark of* Terminalia fagifolia *(500 mg/kg) or its aqueous (TFAqF) and hydroalcoholic (TFHAF) partition fractions (250 mg/kg) or carbenoxolone (100 mg/kg) on the gastric wall mucus and nonprotein sulfhydryl (NP-SH) group contents in male rats treated by subcutaneous route with indomethacin are presented in Figure 5. TFEE, TFAqF, and TFHAF showed an effect similar to that of indomethacin, reducing the gastric wall mucus content of the animals treated with this aggressive agent. The treatment of the animals with indomethacin did not produce a significant depletion of the gastric wall nonprotein sulfhydryl (NP-SH) group content, and TFEE, TFAqF, and TFHAF showed no effect on this gastric wall constituent.

### 3.12. Effect of TFEE, TFAqF, and TFHAF on the Gastric Emptying and Bowel Transit in Rats

**Figure 6:**
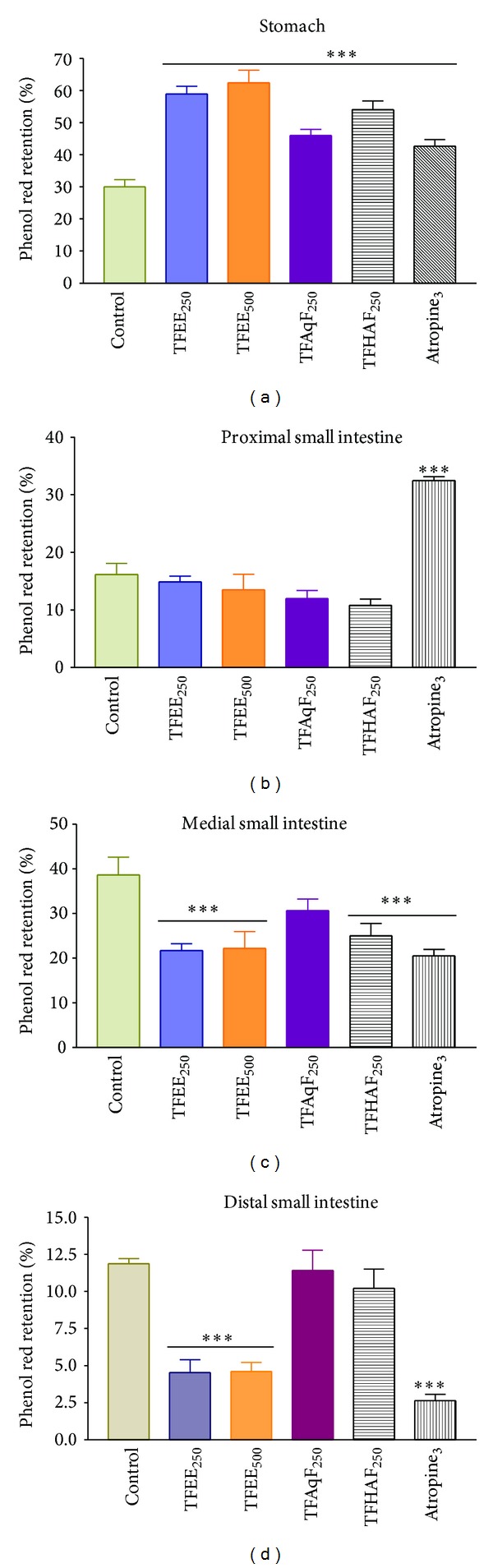
Effect of oral administration of* Terminalia fagifolia* ethanolic bark extract (TFEE, 250 or 500 mg/kg) or its aqueous (TFAqF, 250 mg/kg) and hydroalcoholic (TFHAF, 250 mg/kg) partition fractions or atropine (3 mg/kg) on the gastric emptying of female rats (8 animals/group). Data are presented as the mean ± S.E.M. ***P < 0.01 compared to the respective control group (ANOVA and Dunnett's test).

The percentage distribution of phenol red recovered from the four gastrointestinal segments of treated female rats is detailed in Figure 6. The percentage of dye recovered from the stomach of rats following administration of TFEE at 250 mg/kg (58.9 ± 2.4) and 500 mg/kg (62.4 ± 4.0) was significantly (*P* < 0.01) greater than that observed in control rats (30.0 ± 2.2), indicating that TFEE delays the gastric emptying. The percentage of recovered phenol red was significantly lower (*P* < 0.01) in the medial and distal intestinal segments for animals treated with TFEE at 250 (21.7 ± 1.5 and 4.5 ± 0.8) and 500 mg/kg (22.2 ± 3.8 and 4.6 ± 0.6) compared to the respective control groups (38.7 ± 4.0 and 11.9 ± 0.4). The animals treated with TFAqF showed a significant increase (*P* < 0.01) in the dye gastric content (46.6 ± 2.0), but there were no differences with respect to the dye contents in the small intestinal segments. The animals treated with TFHAF showed a significant increase (*P* < 0.01) in the dye gastric content (54.0 ± 2.8) and a significant decrease ( *P* < 0.01) in the medial small intestinal segment (25.0 ± 2.8), but there were no differences with respect to the dye contents in the proximal (10.8 ± 1.1) and distal (10.2 ± 1.3) small intestinal segments, compared to the respective control groups (16.1 ± 1.9 and 11.9 ± 0.4). Atropine (3 mg/kg) provoked a significant increase (*P* < 0.01) in the dye contents in the gastric (42.6 ± 2.1) and proximal small intestinal segment (32.4 ± 0.7) and a significant decrease (*P* < 0.01) in the medial (20.5 ± 1.5) and distal (2.6 ± 0.4) small intestinal segments.

### 3.13. Effect of TFEE and TFHAE on the Small Intestinal Transit in Mice

The advancement of the charcoal meal along the small intestine of mice was decreased 21% and 62% by the treatment with TFEE at doses of 750 and 1000 mg/kg, respectively, 21%, 30%, and 34% by the treatment with TFHAE at doses of 500, 750, and 1000 mg/kg, respectively, and 60% by the treatment with atropine (3 mg/kg). This result was significantly different (*P* < 0.05) from what was seen in the control group, indicating that TFEE and TFHAE induced an inhibition of the small intestinal transit but only when the extracts were administered at higher doses (Table 4).

### 3.14. Effect of TFEE on Castor Oil-Induced Diarrhea in Mice

**Figure 7:**
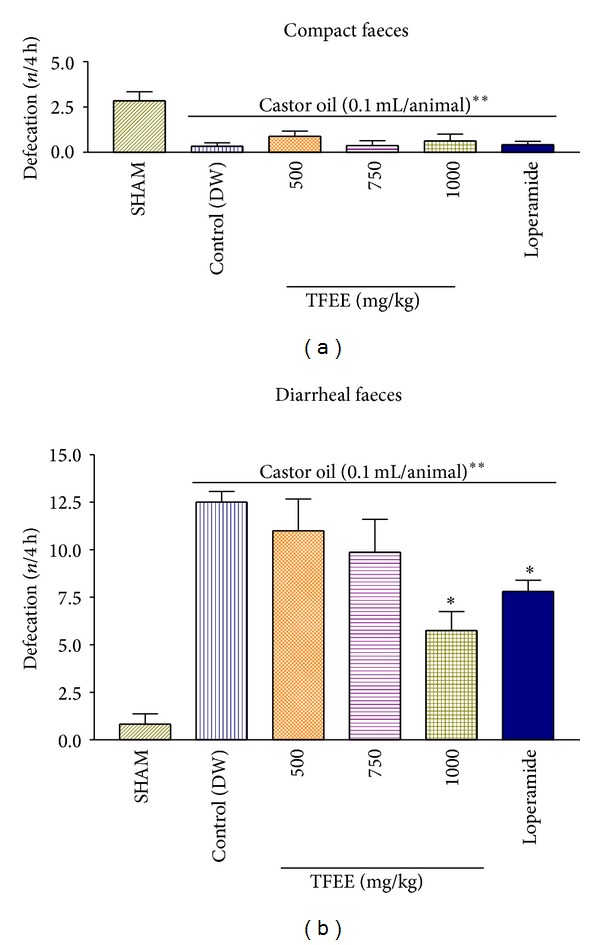
Effect of the oral treatment of mice with different doses of* Terminalia fagifolia* ethanolic bark extract (TFEE) and loperamide (3.5 mg/kg) on the diarrheal activity of castor oil. Data are presented as the mean ± S.E.M. of 8 animals/group. *P < 0.05 compared to the respective castor oil control group treated with distilled water (DW); **P < 0.01 compared to the SHAM group (ANOVA and Dunnett's test).

Using the castor oil-induced diarrhea model on mice, it was observed that the severity of the diarrhea was significantly reduced (*P* < 0.05) by the treatment of the animals with TFEE (1000 mg/kg) and loperamide (3.5 mg/kg), indicating a weakly antidiarrheal activity in the ethanolic extract of this plant (Figure 7).

## 4. Discussion and Conclusion

Peptic ulcer is one of the most common gastrointestinal diseases. This pluricausal illness is a resultant of an interaction and imbalance between aggressive factors—like ethanol, free radicals, hydrochloric acid, ischemia, leukotrienes, NSAIDs, pepsin, and stress—and defensive factors like bicarbonate, mucus, mucosal blood flow, sulfhydryl and enzymatic activity of superoxide dismutase, and catalase [38]. Prostaglandins and nitric oxide (NO) are important factors involved in gastric defense mechanisms through the regulation of acid and alkaline secretion, epithelial fluid, mucus secretion, and mucosal blood flow [43, 49].

The results of this investigation showed that the oral administration of* Terminalia fagifolia* ethanolic bark extract (TFEE) had an antiulcerogenic activity against ethanol-induced gastric ulcer, which was reduced by pretreatment with L-NAME and indomethacin. By intraduodenal route of administration, TFEE showed an antisecretory property but did not change the gastric wall mucus and nonprotein sulfhydryl group content in pylorus ligated rats, indicating a systemic activity after the intestinal absorption of the active constituents of the plant extract. Nevertheless, orally administered TFEE and its aqueous (TFAqF) and hydroalcoholic (TFHAF) partition fractions drastically reduced the mucus layer adhered to the gastric wall of rats treated with ethanol or indomethacin. These results point out that the prostaglandins pathway seems to be only partially involved in the gastroprotective effect of TFEE since the gastric wall mucus was reduced by TFEE but not by carbenoxolone, an agent that enhances the prostaglandins synthesis. Moreover, TFEE delayed gastric emptying and presented a relatively low toxicity and an antioxidant activity similar to that of catechin, used as a comparative standard. Besides these properties, TFEE slightly inhibited the basal and castor oil stimulated small bowel motility, demonstrating a weakly antidiarrheal activity.

The aqueous (TFAqF) and hydroalcoholic (TFHAF) but not the hexanic (TFHEXF) partition fractions of TFEE also presented significant antiulcerogenic activity and delayed gastric emptying, indicating that those properties could be related to the action of polyphenolic compounds like flavonoids or glycosylated flavonoids and saponins present in the bark of* Terminalia fagifolia*.

Ethanol-induced gastric ulcer has been widely used for the evaluation of antiulcerogenic activity of natural products. Ethanol induces ulcers by reducing gastric mucosal blood flow and mucus production in gastric lumen, decreasing endogenous glutathione and prostaglandin levels and increasing ischemia, gastric vascular permeability, acid “back diffusion,” histamine release, efflux of sodium and potassium, influx of calcium, the generation of free radicals, and the production of leukotrienes [38].

Certainly the gastroprotection elicited by the compounds present in the bark of* Terminalia fagifolia* could reflect an inhibition of the gastric acid secretion and an increase in the release of protective substances by the gastric mucosa such as nitric oxide and prostaglandins, since this protection was decreased by pretreatment with L-NAME and indomethacin, which are nitric oxide synthase and cyclooxygenase inhibitors, respectively.

Plants belonging to the botanical family Combretaceae (*Terminalia arjuna*,* T. bellirica*,* T. chebula*,* T. pallida*,* Combretum leprosum*,* C. dolichopetalum,* and* Guiera senegalensis*) have been shown to exhibit antiulcerogenic and gastroprotective activity with the involvement of prostaglandins and nitric oxide [22, 23, 25, 26, 32–35], apart from its ubiquitous antioxidant properties.

There is evidence that reactive oxygen species and free radicals are involved in the etiology and physiopathology of several human diseases, such as gastrointestinal inflammation and gastric ulcer. The potential antioxidant protective effect of natural products on affected tissues, therefore, is a topic of high current interest [50]. Thus, the free radical scavenging activity detected in* Terminalia fagifolia* bark extracts could contribute to their gastroprotective activity.

Antiulcerogenic or gastroprotective activity was detected in plants containing (−)-epicatechin [51–54]. Reimann et al. [55] showed that (+)-catechin (25 mg/kg), given intraperitoneally, prevented the formation of gastric lesions induced by immobilization in female rats. Nevertheless, orally administered (+)-catechin has not presented antiulcer activity in ethanol-induced gastric ulcers in rats [56] and could not be responsible for the detected antiulcerogenic activity of* Terminalia fagifolia* bark extracts. Probably, this activity must be associated with the action of the substances detected on the HPLC analysis with retention time similar to (+)-catechin in TFEE and TFHAF or to (−)-epicatechin in TFAqF. Flavonoids, a large group of polyphenolic compounds, have been reported to exhibit a wide range of biological activity, including antiulcerogenic effect against gastric damaging agents [57–59], and were detected in TFEE and its partition fractions.

Yano et al. [60] showed that, for rats submitted to restraint and water immersion stress, the formation of gastric lesions was markedly accelerated after an increase in gastric motility and that this ulcerogenic effect may be due to the “mechanical rubbing” of the gastric mucosa. According to Cho et al. [61] intermittent vagal electrical stimulation increased the intragastric pressure and induced a 100% incidence of hemorrhagic ulcers in the glandular mucosa of rat stomachs. Hypermotility of the stomach has been considered one of the mechanisms of the ulcerogenic action of indomethacin in rats, probably through microcirculatory disturbances, leading to the increased microvascular permeability and cellular damage [62–65]. The endogenous NO also delays gastric emptying and antral motor activity without affecting gastric myoelectrical activity [66]. Flavonoid-rich fraction of* Maytenus ilicifolia* Mart. ex. Reisseck protects the gastric mucosa of rodents through antisecretory activity and formation of nitric oxide and, at the same time, inhibits the gastric emptying and intestinal motility of mice [67, 68]. These findings substantiate the idea that a delaying in the gastric emptying may be considered a beneficial property of a gastroprotective agent against aggressive factors of the gastric mucosa like ethanol and could also contribute to the antiulcerogenic activity of* Terminalia fagifolia* bark extract.

Our results give partial support to the popular use of the bark of this plant to treat gastrointestinal disorders, such as gastritis and gastric ulcers. Besides its antiulcerogenic and antisecretory activities, the plant extract delayed gastric emptying and presented antioxidant activity. Moreover, the toxicity is relatively low and the intestinal transit is inhibited only with higher doses of the extract. However, the assessment of the quality, safety, and therapeutic efficacy of phytotherapic preparations requires more scientific investigation.

The results found do not allow the full understanding of the mechanisms involved in the pharmacological activities detected in this study. In reality, the data found in relation to the effects of* Terminalia fagifolia* on the mucus layer bound to the wall of the stomach of rats require further study in order to clarify the paradox of the presence of a significant gastroprotector activity in this plant that, at the same time, drastically reduces the mucus layer adhered to the gastric wall.

## Supplementary Material


*Terminalia fagifolia* Mart. and Zucc. Detail of the leaves, bark stem, flowers and fruits. Photo taken by author (P.H.M. Nunes, 2006) at the "Bamboo" Community, Timon-MA, Brazil. Illustration of the gastroprotective activity of the plant bark stem on acute ethanol induced ulcers in the stomach of rats.

## Figures and Tables

**Table 1:** Stoichiometry and reactivity of the DPPH free radical scavenging activity and total phenolic (TPC) and flavonoid (TFC) content for the *Terminalia fagifolia* ethanolic bark extract (TFEE) and the aqueous (TFAqF), hydroalcoholic (TFHAF), and hexanic (TFHEXF) partition fractions of TFEE, catechin, and butylated hydroxytoluene (BHT).

Sample	DPPH scavenging activity		TPC	TFC
	EC_50_ (*μ*g/mL)	ET_50_ (s)	mg GAE/g*	mg RE/g**
TFEE	50.0 ± 3.9	4.5 ± 0.6^a^	452.3 ± 18.1	218.6 ± 2.0
TFAqF	33.9 ± 3.1	8.0 ± 1.9^a^	400.5 ± 9.1^b^	217.5 ± 1.6
TFHAF	31.6 ± 2.0	4.7 ± 1.4^a^	404.1 ± 12.9^b^	222.0 ± 1.9
TFHEXF	>>240^a^	ND	76.6 ± 1.3^b^	21.4 ± 0.5^b^
Catechin	46.3 ± 2.5	51.7 ± 9.7	514.5 ± 6.9^b^	7.4 ± 0.8^b^
BHT	213.8 ± 5.5^a^	ND	356.6 ± 9.3^b^	0.0 ± 0.0

The data represent M ± S.E.M. for a triplicate assay. *P* < 0.01 compared to ^a^catechin or to ^b^TFEE (ANOVA and Tukey's test). ND: not detectable. *mg gallic acid equivalent/g, **mg rutin equivalent/g.

**Table 2:** Mortality of male and female mice treated orally with TFEE, TFAqF, or TFHAF and observed for 14 days.

Treatment (2000 mg/kg, *p*.*o*.)	Mortality (*D*/*T*)*		LD_50_** (mg/kg)
	Male mice	Female mice	
TFEE	0/5	2/5	>2000
TFAqF	—	0/5	
TFHAF	—	1/5	

*D/*T* represents the number of deaths (*D*) among the total (*T*) of tested animals. **OECD, guideline 425.

**Table 3:** Effect of the intraduodenal administration of the *Terminalia fagifolia* ethanolic bark extract (TFEE) and ranitidine on pH and total acidity of gastric secretion and gastric juice volume and on the gastric wall mucus or nonprotein sulfhydryl group contents in four-hour pylorus ligated rats.

Parameter	Vehicle (5 mL/kg)	TFEE (500 mg/kg)	Ranitidine (60 mg/kg)
pH (units)	2.22 ± 0.07	3.76 ± 0.39*	5.80 ± 0.66*
Volume (mL)	3.02 ± 0.17	1.18 ± 0.21*	1.48 ± 0.22*
Total acidity (*μ*Eq/h)	69.77 ± 5.51	13.19 ± 4.33*	5.83 ± 3.13*
Gastric wall mucus (*μ*g/g)	36.07 ± 4.42	34.47 ± 2.83	38.97 ± 2.32
Nonprotein SH (*μ*M/g)	48.17 ± 1.80	46.11 ± 2.45	42.44 ± 3.37

The data represent the mean ± S.E.M. of groups of 6–8 animals. *P < 0.05 (ANOVA and Dunnett's test) compared to the respective control group.

**Table 4:** Effect of the oral treatment of mice with different doses of the *Terminalia fagifolia* ethanolic (TFEE) or hydroalcoholic (TFHAE) bark extract or atropine on the small intestinal transit of an aqueous suspension of charcoal.

Treatment	Dose (mg/kg)	*N*	Advancement of the charcoal (%)	Inhibition (%)
Control	—	12	67.57 ± 2.02	0

TFEE	500	8	62.99 ± 2.84	7
	750	7	53.47 ± 2.37*	21
	1000	7	25.41 ± 6.42***	62

TFHAE	500	7	53.56 ± 3.96*	21
	750	8	47.12 ± 4.36**	30
	1000	7	44.68 ± 4.50**	34

Atropine	3	8	27.35 ± 3.49***	60

Data are presented as the mean ± S.E.M. *P < 0.05; **P < 0.01 and ***P < 0.001 compared to the control group (ANOVA and Tukey's test).
