# Supplementary figures and images for: Gastric Antiulcerogenic and Hypokinetic Activities of Terminalia fagifolia Mart. & Zucc. (Combretaceae)
Source: Biomed Res Int. 2014 May 12;2014:261745. doi: 10.1155/2014/261745 (PMC4036414; doi:10.1155/2014/261745)

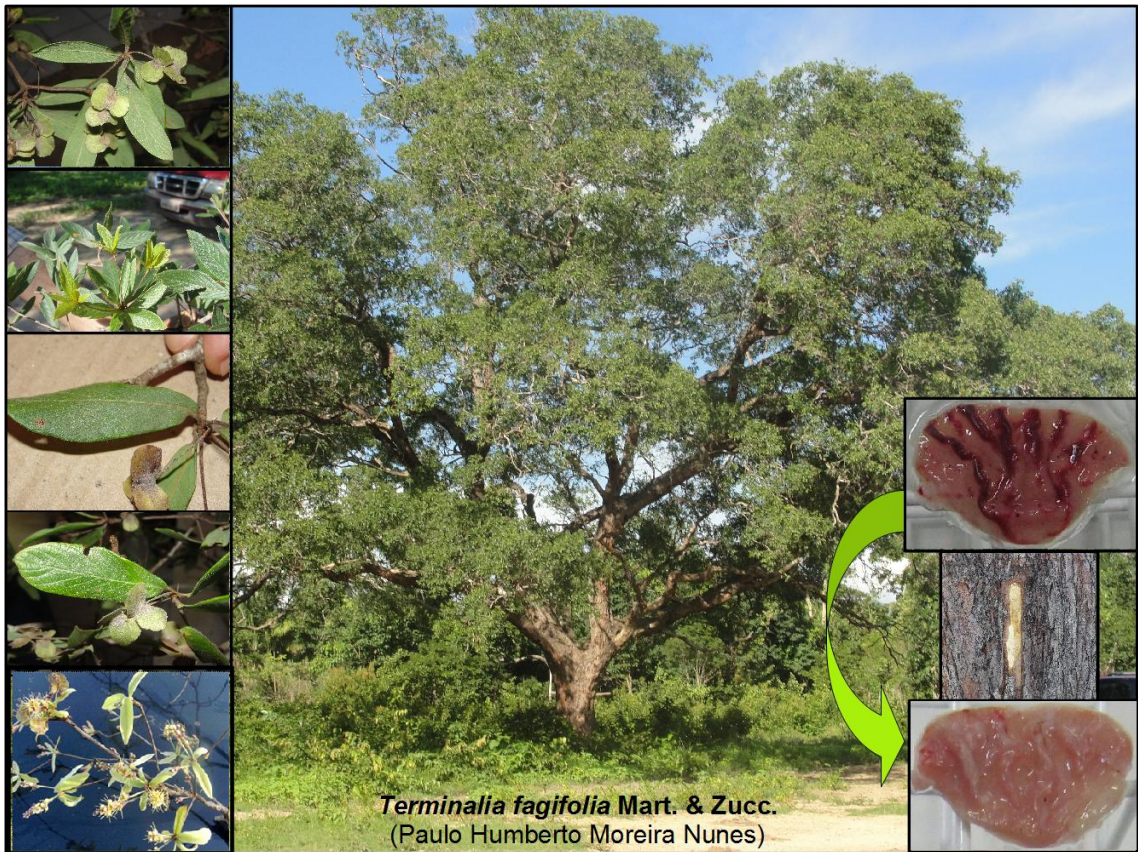

Supplement: Supplementary file 1 — Terminalia fagifolia Mart. and Zucc. Detail of the leaves, bark stem, flowers and fruits. Photo taken by author (P.H.M. Nunes, 2006) at the "Bamboo" Community, Timon-MA, Brazil. Illustration of the gastroprotective activity of the plant bark stem on acute ethanol induced ulcers in the stomach of rats. [file 261745.f1.pdf]
